# Influenza vaccine failure in the tropics: a retrospective cohort study of waning effectiveness

**DOI:** 10.1017/S0950268820002952

**Published:** 2020-12-02

**Authors:** B. E. Young, T. M. Mak, L. W. Ang, S. Sadarangani, H. J. Ho, A. Wilder-Smith, T. Barkham, M. Chen

**Affiliations:** 1 National Centre for Infectious Diseases, Singapore; 2 Lee Kong Chian School of Medicine, Nanyang Technological University, Singapore; 3 National Public Health Laboratory, National Centre for Infectious Diseases, Singapore; 4 National Public Health and Epidemiology Unit, National Centre for Infectious Diseases, Singapore; 5 Department of Clinical Epidemiology, Office of Clinical Epidemiology, Analytics, and Knowledge, Singapore; 6 London School of Hygiene and Tropical Medicine, London, UK; 7 Heidelberg Institute of Global Health, University of Heidelberg, Germany; 8 Department of Laboratory Medicine, Tan Tock Seng Hospital, Singapore; 9 Department of Microbiology and Immunology, Yong Loo Lin School of Medicine, National University of Singapore, Singapore; 10 Saw Swee Hock School of Public Health, National University of Singapore, Singapore

**Keywords:** Effectiveness, influenza, tropics, vaccine, waning

## Abstract

Influenza vaccine effectiveness (VE) wanes over the course of a temperate climate winter season but little data are available from tropical countries with year-round influenza virus activity. In Singapore, a retrospective cohort study of adults vaccinated from 2013 to 2017 was conducted. Influenza vaccine failure was defined as hospital admission with polymerase chain reaction-confirmed influenza infection 2–49 weeks after vaccination. Relative VE was calculated by splitting the follow-up period into 8-week episodes (Lexis expansion) and the odds of influenza infection in the first 8-week period after vaccination (weeks 2–9) compared with subsequent 8-week periods using multivariable logistic regression adjusting for patient factors and influenza virus activity. Records of 19 298 influenza vaccinations were analysed with 617 (3.2%) influenza infections. Relative VE was stable for the first 26 weeks post-vaccination, but then declined for all three influenza types/subtypes to 69% at weeks 42–49 (95% confidence interval (CI) 52–92%, *P* = 0.011). VE declined fastest in older adults, in individuals with chronic pulmonary disease and in those who had been previously vaccinated within the last 2 years. Vaccine failure was significantly associated with a change in recommended vaccine strains between vaccination and observation period (adjusted odds ratio 1.26, 95% CI 1.06–1.50, *P* = 0.010).

## Introduction

Development of an influenza vaccine began soon after the virus was first isolated from humans during the winter epidemic of 1932–1933 [1]. While vaccine design has evolved from whole-virus to less reactogenic split-virion and subunit vaccines, they continue to rely on generating antibodies against the highly variable haemagglutinin (HA) head for effectiveness [2].

The duration of protection after vaccination appears to be relatively short [3, 4]. This was illustrated in a human challenge study with A/H1N1 conducted in 102 long-term adult male residents of Yipsilanti State Hospital by *Francis et al*., in 1942 [5]. Fever >100°F (37.8 °C) developed in half (18/36) of the unvaccinated controls, 32.1% (9/28) of the group vaccinated 4 1/2 months earlier, but only 15.8% (6/38) of individuals vaccinated 2 weeks previously.

Analysis of data collected from clinical trials and test-negative design (TND) case−control studies have also indicated vaccine effectiveness (VE) wanes in the months after vaccination [6–8]. The largest study to date, a TND study of 49 272 individuals in California estimated the odds for influenza infection increased by 16% for each additional 28-days since vaccination, reaching an odds ratio (OR) of 2.06 (95% confidence interval (CI) 1.69–2.51) more than 154 days after vaccination [9, 10].

TND studies have become the preferred observational study design for assessing influenza VE. However, they typically enrol a large proportion of unvaccinated individuals and hence are an indirect method of measuring changes in VE with time. TND studies have also mainly enrolled outpatients with mild infections, have not been powered to identify patient risk factors for waning VE and are limited by inherent biases [11, 12].

The duration of protection following influenza vaccination is important for optimising the timing of vaccine administration in temperate regions but is of particular interest in tropical and sub-tropical climates where influenza activity is not confined to a relatively short winter season [13]. In these regions, multiple annual outbreaks and continuous year-round transmission are frequently observed [14]. The timing of outbreaks can also be highly variable, which complicates determining the best time of year to vaccinate [15]. Limited data are available from tropical countries, but TND studies from Thailand, Kenya and Singapore indicate VE continues to decline throughout the year after vaccination [16–18].

We conducted a cohort study of influenza vaccine recipients and measured changes in the risk of severe influenza infection with time post-vaccination in a tropical setting with year-round influenza virus activity.

## Methods

We conducted a retrospective cohort study in Singapore. All influenza vaccinations administered to Singapore residents aged ⩾21 years at National Healthcare Group (NHG) facilities between 1 January 2013 and 22 July 2017 were identified from a regional vaccination database. The NHG cluster operates public healthcare facilities in Singapore, including Tan Tock Seng Hospital (TTSH) a 1500 bedded tertiary hospital and six polyclinics (government-funded primary care clinics) which serve 834 000 patients annually [19].

Vaccination records were matched with electronic medical records of each individual's demographics, co-morbidities and hospitalisations at TTSH. Vaccination records were also matched with TTSH laboratory records of influenza polymerase chain reaction (PCR) tests conducted between 1 January 2013 and 30 September 2017. All influenza PCRs were conducted as part of routine clinical care and used the *ab*TES Flu qPCR Kit (AitBiotech, Singapore). This is a 4-plex real-time assay that tests for influenza A with H3N2 and H1N1-specific subtyping and influenza B. Lineage (B/Victoria; B/Yamagata) is not available with this assay.

Influenza vaccine failure was defined as a positive influenza PCR within 2–49 weeks (14–349 days inclusive) after vaccination and associated with admission to TTSH. Vaccine failure was tagged to the most recent influenza vaccine received prior to infection.

Time following vaccination was split in a Lexis expansion into six sequential 8-week observation periods per subject. The first observation period, weeks 2–9 after vaccination (days 14–69), was the reference to which subsequent observation periods were compared. These periods covered weeks 10–17, 18–25, 26–33, 34–41 and 42–49 post-vaccination. Each vaccination was followed for 49 weeks until death, PCR-confirmed influenza, another vaccination (at which time a new observation is started) or the study end date (30 September 2017).

Because of the changing pattern of influenza virus activity over the course of the study, seasonal influenza activity was estimated for each study participant for each 8-week observation period. Virus activity was estimated from the number of positive influenza PCRs recorded at TTSH for that observation period as a proportion of infections for the total duration of the study.

Predictors of influenza vaccine failure were identified by multivariable logistic regression of the whole cohort. Subsequently, the odds of influenza infection in each observation period compared with the first period and adjusted for predictors of vaccine failure was calculated (adjusted odds ratio (aOR)). VE relative to the first period was calculated by (1/aOR) × 100. We conducted sub-group analysis to explore the relative impact of the main predictors of influenza vaccine failure on changes in VE with time since vaccination. Observation periods were additionally modelled as a continuous variable to estimate the overall trend.

Influenza infections during periods where a recommended vaccine strain had not changed for more than 1 year and where the infected individual had received that vaccine were identified and the sample was retrieved for sequencing. RNA was extracted and whole genome amplification was achieved by reverse transcription PCR (RT-PCR) using Superscript^®^ III One-Step RT-PCR System with Platinum^®^ Taq DNA polymerase (Invitrogen) [20]. RT-PCR products were pooled, libraries were prepared using the Nextera XT DNA lkit and sequenced on the MiSeq instrument (Illumina). HA sequences were processed and assembled using a customised pipeline of open-source software programs (unpublished). Phylogenetic analysis was performed using MEGA 6 (https://www.megasoftware.net) using maximum likelihood test with 1000 bootstrap replicates. Key amino acid changes were identified using FluServer [21].

Statistical analysis was performed using R version 3.6.1 (R Foundation for Statistical Computing, Vienna, Austria). Statistical significance was assumed with *P* < 0.05. For the initial multivariable logistic regression model of influenza vaccine failure, the Bonferroni correction was applied to account for multiple comparisons when selecting variables to include in the logistic regression model. Variables were removed by backwards elimination. Statistical tests included Student's *t* test for continuous variables and chi-square test for categorical variables. This study was approved by the institutional ethics committee (Ref: 2017/00691) with a waiver of informed consent from participants. Data analysed in this paper are available from the corresponding author on reasonable request.

## Results

### Influenza vaccinations

**Fig. 1.**
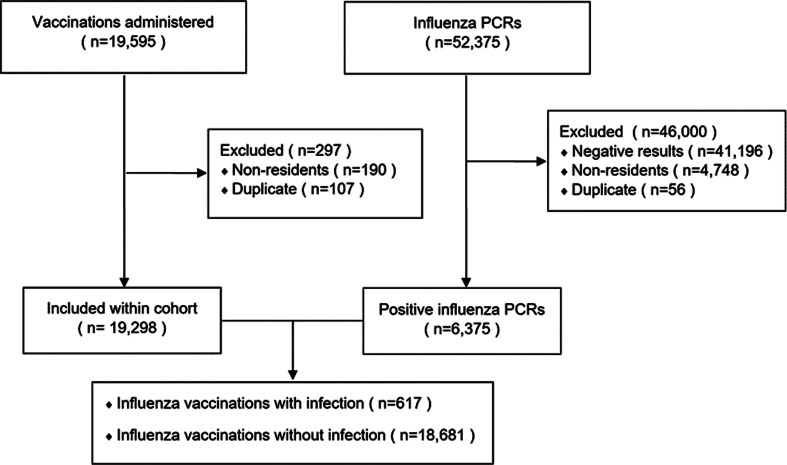
Flow diagram of study.

A total of 19 298 vaccinations met study inclusion criteria and were administered to 11 462 Singapore residents (Fig. 1). All were standard-dose (15 mcg HA per strain) egg-cultured inactivated influenza vaccine without adjuvant. Strain composition reflected contemporary World Health Organization (WHO) recommendations and changed every 6 months to the Northern Hemisphere or Southern Hemisphere vaccine (Supplementary Table 1). Vaccines were either subunit or split-virion and from a range of manufacturers over the course of study. The majority of vaccines administered were trivalent (95.8%). This was replaced by the quadrivalent vaccine with two B strains in April 2017.

**Fig. 2.**
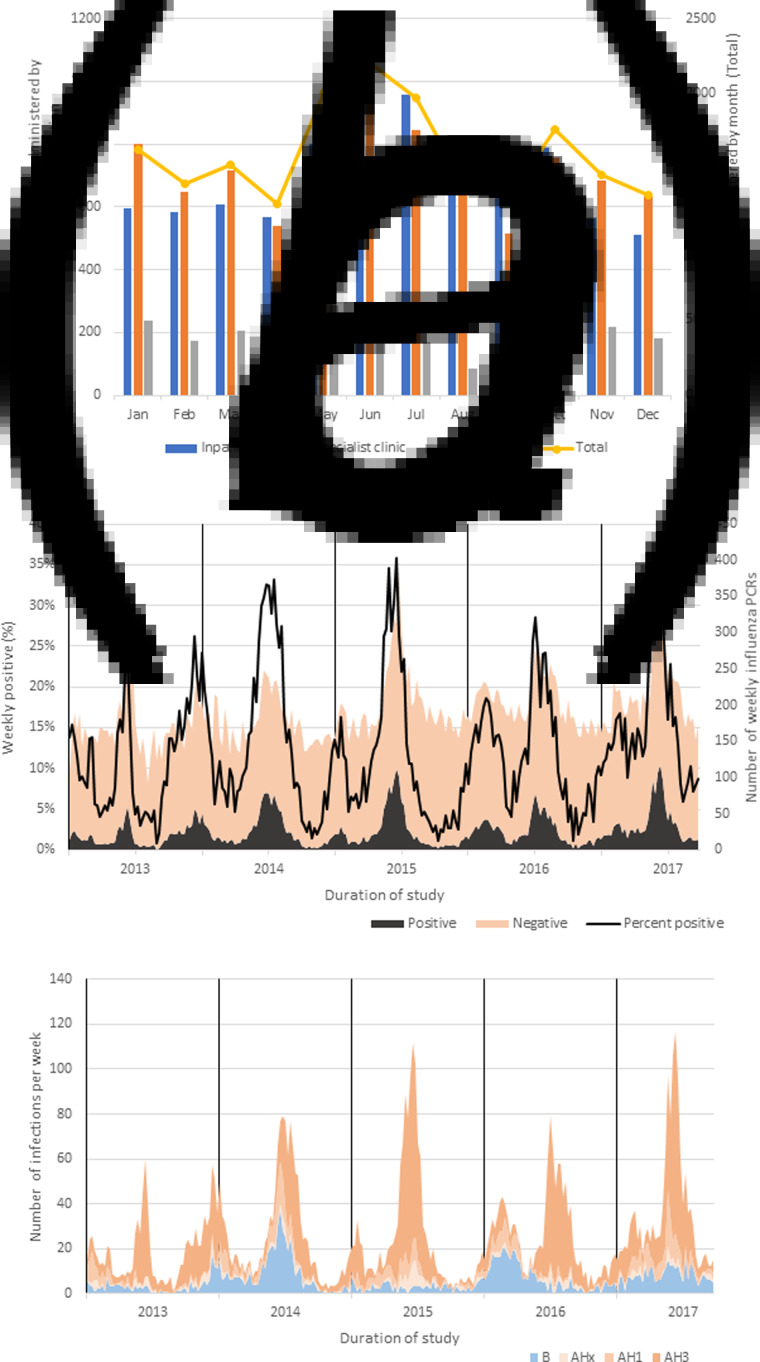
(A) Number of influenza vaccines administered by month and vaccination location (all years combined); (B) weekly number of unique influenza PCRs conducted at Tan Tock Seng Hospital over the course of the study with a positive or negative result; (C) weekly influenza subtype/type results reported from positive influenza PCRs. AHx, Influenza A, subtype undetermined.

Vaccines were administered throughout the year (Fig. 2A). The weekly number of vaccines administered increased from an average of 57 per week in 2013 to 99 in 2017 (*t* test, *P* < 0.0001, Supplementary Fig. 1). The majority of vaccines were administered at the hospital, either during an inpatient admission (42.6%) or at a specialist outpatient clinic (45.5%), while 11.8% were administered at NHG polyclinics.

### Influenza PCR

Of the 52 375 unique influenza PCR records over the study period, 6375 were positive and met study inclusion criteria. Infections were detected year-round, but with biannual epidemics (Fig. 2B). There was little variation in the number of negative influenza tests between influenza epidemic and inter-epidemic periods, however, there was a significant increase in the number of tests per week over the course of the study (*t* test, *P* < 0.0001, Supplementary Fig. 2). The majority of influenza infections were type A (77.0%), with subtype AH3 comprising 82.0% and AH1 18.0% (Fig. 2C). No avian influenza infections (e.g. AH5, AH7) were detected in Singapore over the course of the study (Supplementary Fig. 3).

### Vaccine failure

In total, 652 influenza infection were identified within 14–365 days after administration of the 19 298 vaccines. After excluding 11 infections which were not associated with hospital admission and 24 which occurred from week 50 to 52, 617 infections occurred during the weeks 2–49 after vaccination, a vaccine failure rate of 3.2%.

Subject attrition due to death or end of follow-up was stable across the six observation periods after influenza vaccination, while re-vaccination rates steadily increased (Table 1). Participants remaining in the study at later observation periods were of a similar age to the baseline period, but had fewer co-morbidities and were less likely to have been admitted in the year prior to vaccination. Increasing age, higher Charlson's co-morbidity score, presence of chronic pulmonary disease, history of hospital admission in the preceding year and receipt of influenza vaccination received less than 2 years previously were significantly associated with vaccine failure (Supplementary Table 2).

**Table 1.** Summary data by individual observation periods post-vaccination

Period	1	2	3	4	5	6
Weeks post-vaccination	2–9	10–17	18–25	26–33	34–41	42–49
Number of observation periods	19 298	17 747	16 451	15 275	14 070	12 762
Year of vaccination, *n* (%)						
2013	3027 (15.7)	2975 (16.8)	2907 (17.7)	2852 (18.7)	2852 (20.3)	2852 (22.3)
2014	3300 (17.1)	3224 (18.2)	3157 (19.2)	3027 (19.8)	3027 (21.5)	3027 (23.7)
2015	5154 (26.7)	5006 (28.2)	4844 (29.4)	4660 (30.5)	4660 (33.1)	4660 (36.5)
2016	4802 (24.9)	4661 (26.3)	4527 (27.5)	4396 (28.8)	4396 (31.2)	4396 (34.4)
2017	3015 (15.6)	1881 (10.6)	1016 (6.2)	340 (2.2)	340 (2.4)	340 (2.7)
Change in vaccine strain^a^, *n* (%)						
Any	579 (3.0%)	3126 (17.6)	5492 (33.4)	7789 (51.0)	9064 (64.4)	10 375 (81.3)
AH3	255 (1.3%)	1776 (10.0)	3355 (20.4)	5281 (34.6)	6526 (46.4)	7952 (62.3)
AH1	167 (0.9%)	846 (4.8)	1238 (7.5)	1228 (8.0)	1276 (9.1)	1229 (9.6)
B	326 (1.7%)	1746 (9.8)	3306 (20.1)	5020 (32.9)	6285 (44.7)	7695 (60.3)
Attrition						
Re-vaccination, *n* (%)	76 (0.39%)	97 (0.55%)	172 (1.0%)	326 (2.1%)	389 (2.8%)	1242 (9.7%)
All-cause mortality, *n* (%)	347 (1.8%)	298 (1.7%)	279 (1.7%)	251 (1.6%)	216 (1.5%)	176 (1.4%)
End of follow-up, *n* (%)	-	1023 (5.3%)	787 (4.4%)	633 (3.8%)	522 (3.4%)	592 (4.2%)
Influenza infection						
Number of infections, *n* (%)	105 (0.544)	114 (0.642)	92 (0.559)	106 (0.694)	111 (0.789)	89 (0.697)
Mean risk of infection compared with all time periods^b^						
All	0.983	0.908	0.963	1.065	1.083	1.031
AH3	1.024	0.895	0.949	1.044	1.063	1.052
AH1	0.917	0.928	0.994	1.103	1.102	0.997
B	0.907	0.929	0.980	1.100	1.125	1.006
Patient characteristics						
Age, years, mean (s.d.)^c^	71.9 (14.7)	71.8 (14.7)	71.8 (14.7)	71.7 (14.7)	71.7 (14.6)	71.7 (14.7)
>85 years	19.6%	19.3%	19.1%	18.9%	18.8%	18.7%
>65 years	71.3%	71.3%	71.2%	71.1%	71.3%	71.0%
Male	56.7%	56.7%	56.8%	56.8%	56.5%	56.7%
Charlson comorbidity index, mean (s.d.)	2.93 (2.53)	2.90 (2.52)	2.86 (2.50)	2.82 (2.48)	2.79 (2.47)	2.76 (2.46)
Chronic pulmonary disease	44.6%	44.6%	44.1%	43.9%	43.6%	42.9%
Hospitalisation in year prior to vaccination	66.3%	65.7%	64.9%	64.5%	64.5%	64.0%
Vaccination in the 2 years prior to index vaccine	45.5%	45.1%	44.8%	44.7%	44.1%	43.5%

a Change in vaccine strain is defined as the availability of vaccine with a new strain composition between the date of vaccination and date of onset of the observation period.

b Estimated average risk of influenza for that observation period compared with all 95 603 time periods under observation. Numbers >1 indicate greater than average risk.

c Mean age and age categories adjusted from date of vaccination to onset of the observation period. s.d., standard deviation.

### VE with time since vaccination

**Fig. 3.**
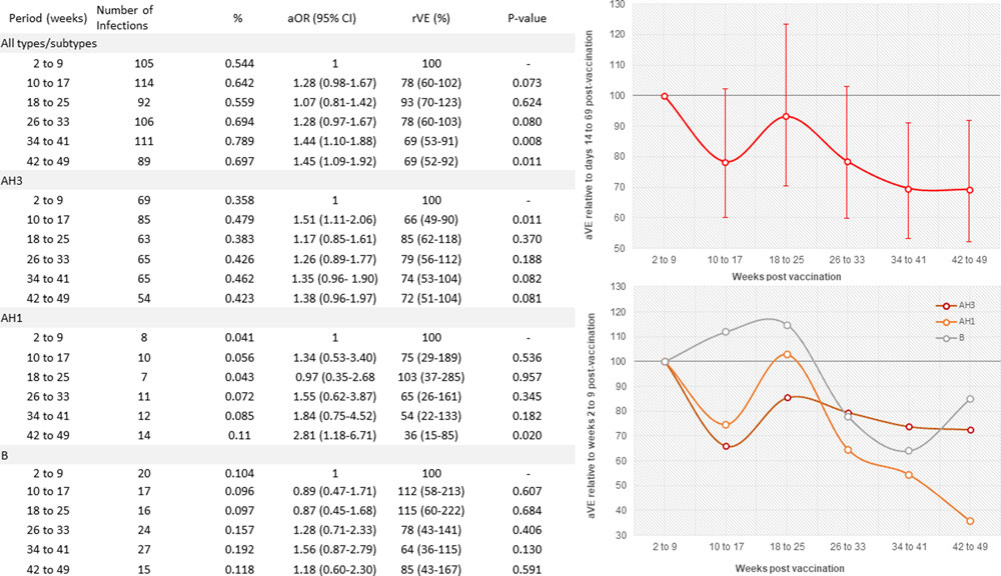
Adjusted influenza vaccine effectiveness (aVE) for influenza infection overall and by influenza type/subtype for each time period relative to the first period (weeks 2–9 after vaccination). The estimates were adjusted for age, sex, ethnicity, chronic pulmonary disease, hospital admission in the previous year, previous influenza vaccination in the 2 years before the index date of vaccination and influenza virus activity during that time period. The error bars indicate 95% confidence interval (CI). aOR, adjusted odds ratio.

Despite a high infection rate in the 2–9 weeks after vaccination, the aOR for any influenza infection relative to this period was significantly higher for the last two observation periods, weeks 34–41 and 42–49 (Fig. 3). There was a significant linear trend of increased odds of influenza infection per period (aOR 1.07, 95% confidence interval (CI) 1.02–1.12). The trend was similar when analysed by influenza type/subtype.

**Fig. 4.**
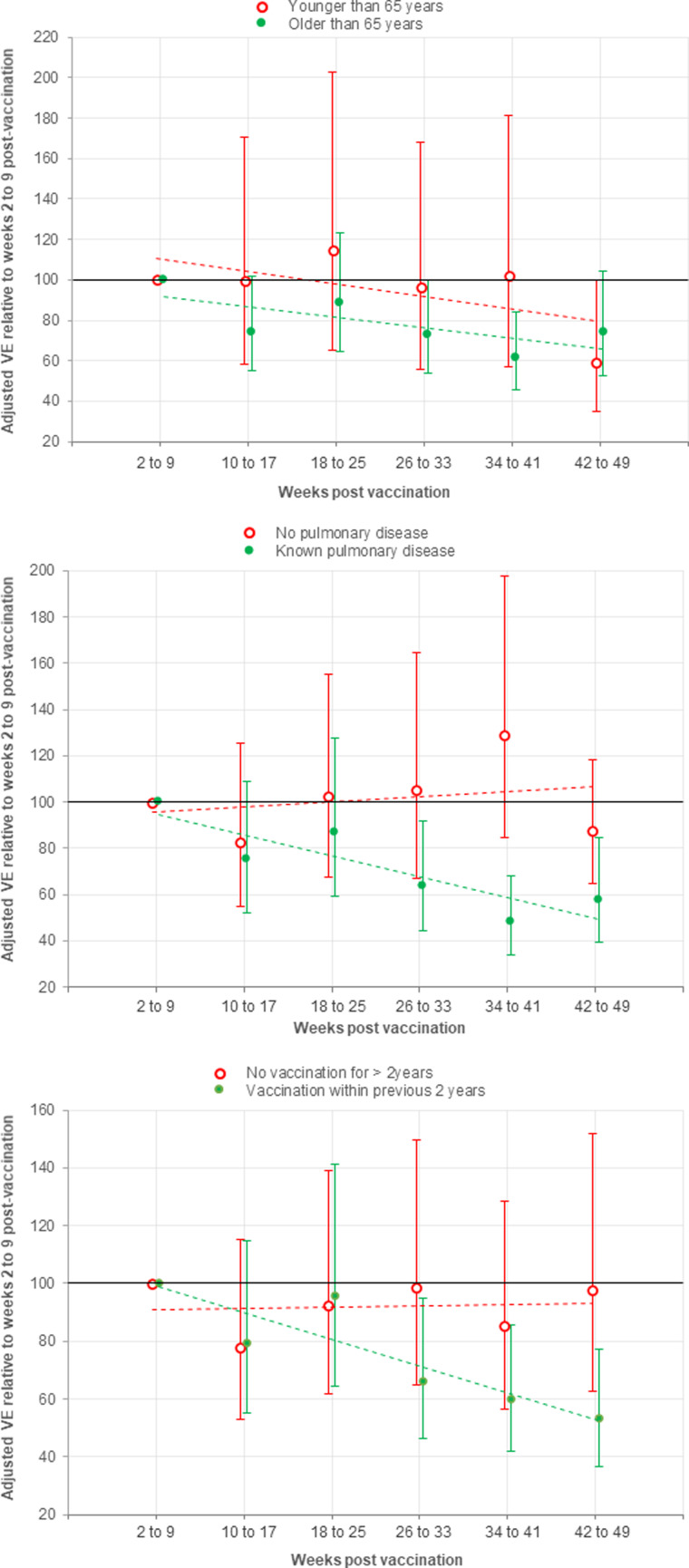
Adjusted vaccine effectiveness against influenza infection by subgroup for each time period relative to the first period (weeks 2–9 after vaccination), with a linear trend line. Estimates were adjusted for age, sex, ethnicity, chronic pulmonary disease, hospital admission in the previous year and previous influenza vaccination in 2 years before the index date of vaccination. The error bars indicate 95% confidence interval (CI). A significant trend of a declining VE with time since vaccination was only observed in those sub-groups at higher risk for influenza vaccine failure (<65 years *P* = 0.104, ⩾65 years *P* = 0.0216; no chronic pulmonary disease *P* = 0.609, chronic pulmonary disease *P* = 0.00012; No vaccination for >2 years *P* = 0.901, previous vaccination *P* < 0.0001).

In the sub-groups at highest risk for influenza there was a significantly increased odds of influenza infection with time since vaccination (Fig. 4). These risk factors also had additive effects, such that with an increasing number of risk factors (age ⩾65 years, chronic pulmonary disease, vaccination in previous 2 years), the incidence of hospitalisation with influenza also increased with time and also the rate of waning VE (Supplementary Fig. 4). The number of influenza vaccines received in the previous 2 years was significantly associated with both an increasing risk of influenza infection and waning VE (Supplementary Fig. 5).

### Effect of changing vaccine strain recommendations

The recommended strain composition of the trivalent vaccine changed five times over the course of the study, with changes to seven vaccine strains: three times for influenza AH3 (new strains Southern Hemisphere (SH) 2014, SH 2015, SH 2016), three times for B (new strains Northern Hemisphere (NH) 2013/14, SH 2015, SH 2016) and once for AH1 (SH 2017).

The proportion of observation periods which occurred after the vaccine strain had changed but the individual had not been re-vaccinated, increased with time since vaccination (Table 1). Overall, 26.3% of AH3, 6.3% of AH1 and 25.5% of B observation periods followed a change in the vaccine strain between SH and NH vaccine or vice versa. The observation periods following a change in vaccine strain were strongly associated with higher AH3 and AH1 influenza virus activity (both *P* < 0.0001), but significantly lower influenza B activity (*P* < 0.0001). After adjusting for other predictors of influenza vaccine failure, including virus activity, change in vaccine strains was associated with an increased odds of influenza infection (aOR 1.26, 95% CI 1.06–1.50, *P* = 0.010). Of note, for the subgroup of individuals for which there was no change in vaccine strain recommendations between reference and observation period, VE still waned with time since vaccination (aOR, 1.09, 95% CI 1.01–1.171, *P* = 0.030).

### Effect of antigenic drift on VE

Vaccine strain recommendations were stable for longer than 1 year for AH1 infections from January 2013 to April 2017 and for AH3 from May 2016 to October 2017. A total of 62 AH3 clinical samples from individuals receiving the A/Hong Kong/4801/2014 vaccine strain were sequenced. All 62 sequences belonged to the 3C.2a clade and contained the K160T mutation, which resulted in the potential gain of a glycosylation site within antigenic site B (Supplementary Fig. 6). No increase in mutations was observed with increasing time from vaccination (Table 2). A/H1N1 HA sequences were successfully retrieved from 14 vaccinated individuals. All sequences belonged to clade 6B, with only one clinical specimen bearing the G155E mutation that was associated with reduced recognition by antisera against A/California/7/2009 (Supplementary Fig. 7).

**Table 2.** Number of mutations compared to influenza vaccine strain A/HongKong/4801/2014 by period post-vaccination

No. of mutations compared to vaccine strain	No. of HA sequences		
	Period 1–3 (2–26 weeks)	Period 4–6 (27–49 weeks)	Total
7	2	1	3
9	1	1	2
10	1	0	1
11	3	3	6
12	7	1	8
13	10	12	22
14	7	7	14
15	1	4	5
17	0	1	1
Total samples	32	30	62
Average no. of mutations	12.3	13.1	12.7

32 of the HA sequences were from patients with infection within 181 days post-vaccination and 30 of the HA sequences were from patients with infection within 182–349 days post-vaccination. Number of mutations was not significantly different between the two time periods (*t* test, mean difference −0.79 (95% CI −1.75 to 0.18) *P* = 0.107). HA, haemagglutinin.

## Discussion

In a tropical country where the influenza virus circulates year-round, the effectiveness of the standard-dose vaccine at preventing hospital admission with influenza infection declined significantly in the months following vaccination. This decline was most evident in individuals at the highest risk for complications associated with influenza, including older adults and those with chronic pulmonary disease. It is of concern that having received more than one vaccine in a 2-year period was associated with reduced VE and faster waning of protection. The results of this study offer insights into the optimal timing of influenza vaccination in temperate climates and for the 40% of the global population residing in the tropics.

Change in influenza VE after vaccination is the consequence of two main factors: firstly, the initial immune response to vaccination and subsequent waning of vaccine-induced protective immunity and secondly, antigenic drift that diminishes the match between the vaccine and circulating strains [22].

The most readily observable antigenic drift occurs when vaccine strains are updated between the NH and SH winters (and vice versa). As a consequence of biannual changes to vaccine strain composition, an individual may be inadequately protected against circulating strains despite receiving annual vaccination. The data from our study indicate that change in vaccine strains is a risk factor for vaccine failure regardless of how recently the prior vaccine was administrated. While such a result is not surprising, to our knowledge evidence in this context has not previously been presented and suggests that in tropical countries when there is a change in recommended vaccine strain, individuals at highest risk of complications should be re-vaccinated regardless of when they received the previous vaccine [23].

Antigenic drift is also expected to result in increasing antigenic mismatch between vaccine and circulating virus *before* vaccine strains are updated. For example, an analysis of AH3 sequences from clinical samples in Thailand described increasing divergence of circulating strains from the 2016 vaccine strain with time [24]. Predicted VE declined from 74% in 2016 to 48% in 2017. Although limited by the number of AH3 samples sequenced, we did not find a significant association between antigenic drift and time since vaccination after matching by the week of infection. However, a comprehensive assessment would require understanding what was circulating in the community at the same time.

Antigen mismatch is not, however, an all or none phenomenon. Antigenic cartography maps how antibody to the HA or neuraminidase of one influenza strain binds to drifted strains with lower affinity, indicating higher titres would be required for protection against infection [25, 26]. Haemagglutination inhibition (HI) titres clearly wane with time following influenza vaccination or infection and are unlikely to persist year-round in older adults [27]. In individuals with diminished immune responses to vaccination – likely to include older adults and those who have received repeated influenza vaccination – waning of protective antibody will thus affect the duration of VE against matched and mismatched circulating virus.

The effects of mismatch and waning immunity have two potential solutions. A number of influenza vaccines which offer enhanced immunogenicity to the standard-dose are available: the recombinant influenza vaccine (RIV), high-dose vaccine (IIV-HD) and vaccines containing the MF-59 adjuvant (aIIV). Clinical trials conducted in temperate climates have also demonstrated that RIV and IIV-HD offer superior vaccine efficacy compared to the standard dose vaccine [28, 29]. Evidence that these vaccines will extend the duration of clinical protection to year-round is not currently available. Although these vaccines clearly offer superior HI titres at seroconversion, there is little data currently available of HI titres 180 or 360 days after vaccination [30]. It may be that these vaccines offer a short-term boost to titres, which then decay more rapidly. Again, these issues are likely to be most significant in vulnerable groups such as older adults and those with co-morbidities.

An alternative to these ‘enhanced’ vaccines is to administer the standard dose twice a year. Biannual vaccination with the same vaccine strains has been shown to boost HI titres against AH3 and AH1 in a clinical trial conducted in older adults in Singapore [31]. Whether the higher HI titre is substantial enough to reduce influenza infections and whether this approach is cost-effective, needs to be demonstrated.

Another question is whether VE wanes at different rates with different influenza subtypes or strains [32]. The immune consequences of influenza infection during early childhood persist lifelong and may result in a birth cohort effect on the duration of VE [33]. This study was unable to address this adequately due to the relatively small number of infections. VE appeared to wane faster with AH1 compared to AH3, which is consistent with lower AH1 HI titres at seroconversion and after 6 months reported from the study of biannual vaccination [31]. However, this finding may also reflect the lower *absolute* effectiveness of the vaccine against AH3 infection compared to AH1. It is difficult to detect waning effectiveness if the vaccine is suboptimally effective to start with.

This study has a number of limitations. It is a retrospective, observational study with vaccines administered – and infections detected – year-round. While there is the possibility of incorrectly coded administrative data a previous study conducted in 2016 at TTSH indicated there was no significant difference in the extracted Charlson's comorbidity score compared with manual chart review [34]. Some data, such as body habitus or smoking history was not available, but may be important predictors of VE. The virus activity score accurately predicted the observed number of influenza infections in the cohort (data not shown) but other factors will determine an individual's risk of being exposed to influenza which we were unable to measure. Although we had access to records at NHG, we were not able to capture vaccinations and hospital admissions at other healthcare groups. As the probability of re-vaccination increased with time since index vaccine was received, this omission would be expected to bias against the detection of waning VE. Singapore has historically had a low uptake of the influenza vaccine estimated at 15.2% for adults aged ⩾50 years from a national survey conducted in 2013 [35].

This study examined a cohort that included a large proportion of older adults and those with comorbidities where the burden of influenza is greatest. We only studied severe influenza – i.e. infections associated with hospital admission – but as a result we are not able to distinguish whether the observed waning reflects a change in infection rates with time or a change in the severity of infection [36]. Influenza vaccination has been reported to reduce the severity of breakthrough infection in hospitalised individuals and presumably this protection would also wane with time [37]. This effect may explain why waning VE was more evident in patients with chronic pulmonary disease. Reduced VE with repeated years vaccination may also reflect individuals with a higher burden from co-morbidities – and hence more frequent hospital attendances and opportunities for vaccination. This possibly resulted in confounding due to an unhealthy vaccinee bias to which we had insufficient data to adjust for.

In conclusion, the implications of year-round influenza circulation on vaccination strategies are beginning to be explored. This study provides important data to support the hypothesis that annual standard-dose vaccination is unlikely to provide optimal protection. Exploration of alternative strategies in adults at high risk for severe influenza should be a public health priority.

## Supplementary material

For supplementary material accompanying this paper visit https://doi.org/10.1017/S0950268820002952. click here to view supplementary material

## Presentation

Part of this information in this paper was presented at the 29th European Congress of Clinical Microbiology and Infectious Diseases (13–16 April 2019), Amsterdam, Netherlands (Abstract number O1014).
