# Supplementary material for: Influenza vaccine failure in the tropics: a retrospective cohort study of waning effectiveness
Source: Epidemiol Infect. 2020 Dec 2;148:e299. doi: 10.1017/S0950268820002952 (PMC7801929; doi:10.1017/S0950268820002952)
Supplement: Supplementary file 1 [file S0950268820002952sup001.docx]

**Supplementary Table 1**

WHO influenza vaccine strain recommendations. Highlighted text indicates vaccines in which trivalent vaccine strain recommendation changed. B1 reflects strain recommended for trivalent vaccines. Both B1 and B2 are recommended for quadrivalent vaccines. NH: Northern Hemisphere; SH: Southern Hemisphere

|  | AH1 | AH3 | Clade | B1 | Lineage | B2 |
| --- | --- | --- | --- | --- | --- | --- |
| SH2017 | A/Michigan/45/2015 (H1N1)pdm09 | A/Hong Kong/4801/2014 | 3C.2a | B/Brisbane/60/2008 | Victoria | B/Phuket/3073/2013 |
| NH 2016-17 | A/California/7/2009 (H1N1)pdm09 | A/Hong Kong/4801/2014 | 3C.2a | B/Brisbane/60/2008 | Victoria | B/Phuket/3073/2013 |
| SH2016 | A/California/7/2009 (H1N1)pdm09 | A/Hong Kong/4801/2014 | 3C.2a | B/Brisbane/60/2008 | Victoria | B/Phuket/3073/2013 |
| NH 2015-16 | A/California/7/2009 (H1N1)pdm09 | A/Switzerland/9715293/2013 | 3C.3a | B/Phuket/3073/2013 | Yamagata | B/Brisbane/60/2008 |
| SH2015 | A/California/7/2009 (H1N1)pdm09 | A/Switzerland/9715293/2013 | 3C.3a | B/Phuket/3073/2013 | Yamagata | B/Brisbane/60/2008 |
| NH 2014-15 | A/California/7/2009 (H1N1)pdm09 | A/Texas/50/2012 | 3C.1 | B/Massachusetts/2/2012 | Yamagata | B/Brisbane/60/2008 |
| SH2014 | A/California/7/2009 (H1N1)pdm09 | A/Texas/50/2012* | 3C.1 | B/Massachusetts/2/2012 | Yamagata | B/Brisbane/60/2008 |
| NH 2013-14 | A/California/7/2009 (H1N1)pdm09 | A/Victoria/361/2011 | 3C.1 | B/Massachusetts/2/2012 | Yamagata | B/Brisbane/60/2008 |
| SH2013 | A/California/7/2009 (H1N1)pdm09 | A/Victoria/361/2011 ** | 3C.1 | B/Wisconsin/1/2010 | Yamagata | B/Brisbane/60/2008 |
| NH 2012-13 | A/California/7/2009 (H1N1)pdm09 | A/Victoria/361/2011 | 3C.1 | B/Wisconsin/1/2010 | Yamagata |  |

* A/Texas/50/2012 is an A(H3N2) virus that following adaptation to growth in eggs has maintained antigenic properties similar to the majority of recently circulating cell-propagated A(H3N2) viruses including A/Victoria/361/2011.

** A/Ohio/2/2012, A/Maryland/2/2012, A/South Australia/30/2012, A/Brisbane/1/2012 and A/Brisbane/6/2012 are A/Victoria/361/2011-like viruses

**Supplementary Figure 1:** Weekly number of influenza vaccines administered (all locations combined) with linear trendline and correlation coefficient. Weeks numbered consecutively from Week 1 in Jan 2013 to Week 237 in July 2017

**Supplementary Figure 2:** Weekly number of negative influenza PCRs with linear trendline and correlation coefficient. Weeks numbered consecutively from Week 1 in Jan 2013 to Week 247 in Oct 2017

2013 2014 2015 2016 2017

Year

**Supplementary Figure 3:** Influenza data by type/subtype and week of the 4,571 influenza infections in the community reported by Singapore Ministry of Health to the WHO through FluNet (Influenza A 70.0% [AH3 74.9%, AH1 25.1%]; Influenza B 30.0%). AHx: Influenza A, subtype undetermined.

**Supplementary Table 2:** Summary data and odds ratio of predictors for influenza infection after vaccination from logistic regression analysis.

|  | Flu | No flu | OR (95% CI) | p-value | Multivariable (95% CI) ¶ | p-value |
| --- | --- | --- | --- | --- | --- | --- |
| N | 617 | 18676 |  |  |  |  |
| Year vaccination   - 2013 - 2014 - 2015 - 2016 - 2017 | 19.0  18.8  23.0  28.0  11.3 | 15.6  17.0  26.8  24.8  15.8 | -  -  -  -  - |  |  |  |
| Demographics | | | | | | |
| Sex (% male) | 58.2 | 56.6 | 1.069 (0.910-1.257) | 0.42 | 1.054 (0.894-1.244) | 0.53 |
| Age (Mean, SD) †  - Over 65 years (%) | 73.8 (12.87)  76.2 | 71.7 (14.77)  71.1 | 1.010 (1.004-1.016)  1.301 (1.083-1.573) | 0.00060  0.0057 | 1.011 (1.005-1.018)  - | 0.00053 |
| Ethnicity (%)   - Chinese - Indian - Malay - Other | 77.2  8.2  11.1  3.5 | 76.7  9.4  9.2  4.8 | Ref  0.868 (0.640-1.151)  1.227 (0.944-1.571)  0.738 (0.465-1.110) | Ref  0.34  0.12  0.17 | 0.924 (0.678 (1.232)  1.412 (1.075-1.830)  0.762 (0.479-1.149) | 0.60  0.01  0.22 |
| Co-morbidities | | | | | | |
| Charlson score  (mean, SD) | 3.06 (2.508) | 2.93 (2.529) | 1.022 (0.990-1.053) | 0.17 | - |  |
| Co-morbidities (%)   - Pulmonary - Cardiovascular - Diabetes - Renal - Liver - Malignancy | 58.7  39.1  34.9  7.6  22.7  8.4 | 44.1  36.3  36.0  6.3  22.8  7.4 | 1.791 (1.524-2.107)  1.131 (0.960-1.331)  0.944 (0.798-1.115)  0.994 (0.820-1.199)  1.211 (0.883-1.621)  1.133 (0.839-1.497) | <0.0001  0.14  0.50  0.95  0.22  0.40 | 1.588 (1.343-1.881)  -  -  -  -  - | <0.0001 |
| Hospitalisation in year before vaccination (%) | 71.7 | 66.1 | 1.307 (1.097-1.564) | 0.0030 | 1.327 (1.104-1.602) | 0.0028 |
| Influenza in year before vaccination (%) | 8.5 | 8.4 | 1.016 (0.755-1.340) | 0.911 | - |  |
| Vaccination details | | | | | | |
| Vaccine location (%)   - Hospital - Specialist clinic - Polyclinic | 41.3  47.9  10.8 | 42.7  45.4  11.9 | Ref  1.085 (0.916-1.285)  0.930 (0.703-1.214) | 0.347  0.602 | -  - |  |
| Time from prior influenza vaccination (%)   - <1 year - <2 years - <5 years | 25.4  56.6  64.0 | 18.9  45.2  53.8 | 1.454 (1.207-1.744)  1.556 (1.325-1.828)  1.504 (1.276-1.777) | <0.0001  <0.0001  <0.0001 | -  1.523 (1.283-1.810)  - | -  <0.0001  - |

¶ Adjusted for age, sex, chronic pulmonary disease, hospitalisation in year before vaccination and receipt of two or more influenza vaccines in the previous two years

†Per year

**Supplementary Figure 4:** Influenza vaccine failure stratified by number of risk factors (age ≥ 65 years, chronic pulmonary disease, influenza vaccination in the previous two years), with linear trend line.

| Number of risk factors | Influenza infections /n | Proportion of individuals with vaccine failure | Adjusted OR for influenza (95% CI) † | p-value |
| --- | --- | --- | --- | --- |
| 0 | 29/1700 | 1.71% | Ref | - |
| 1 | 188/7875 | 2.39% | 1.51 (1.04-2.29) | 0.0400 |
| 2 | 207/5914 | 3.50% | 2.29 (1.57-3.46) | <0.0001 |
| 3 | 193/3809 | 5.07% | 3.47 (2.37-5.25) | <0.0001 |

†Adjusted for age, sex, ethnicity, chronic pulmonary disease, hospital admission in previous year, previous influenza vaccination in the 2 years before index date of vaccination, and influenza virus activity during that time period.

**Supplementary Figure 5:** Influenza vaccine failure stratified by number of influenza vaccines received in the two years before study entry, with linear trend line.

| Number of vaccines | Influenza infections /n | Proportion of individuals with vaccine failure | Adjusted OR for influenza (95% CI) | p-value |
| --- | --- | --- | --- | --- |
| 1 (index vaccine) | 270/10517 | 2.57% | Ref | - |
| 2 | 248/6584 | 3.77% | 1.51 (1.26-1.81) | <0.0001 |
| 3 | 92/2133 | 4.31% | 1.75 (1.36-2.25) | <0.0001 |
| 4 or more | 7/64 | 10.94% | 3.96 (1.67-7.92) | 0.0004 |

†Adjusted for age, sex, ethnicity, chronic pulmonary disease, hospital admission in previous year, previous influenza vaccination in the 2 years before index date of vaccination, and influenza virus activity during that time period.

**Supplementary Figure 6:** Phylogenetic tree of A/H3 haemagglutinin gene sequenced from a subset of cases.

**
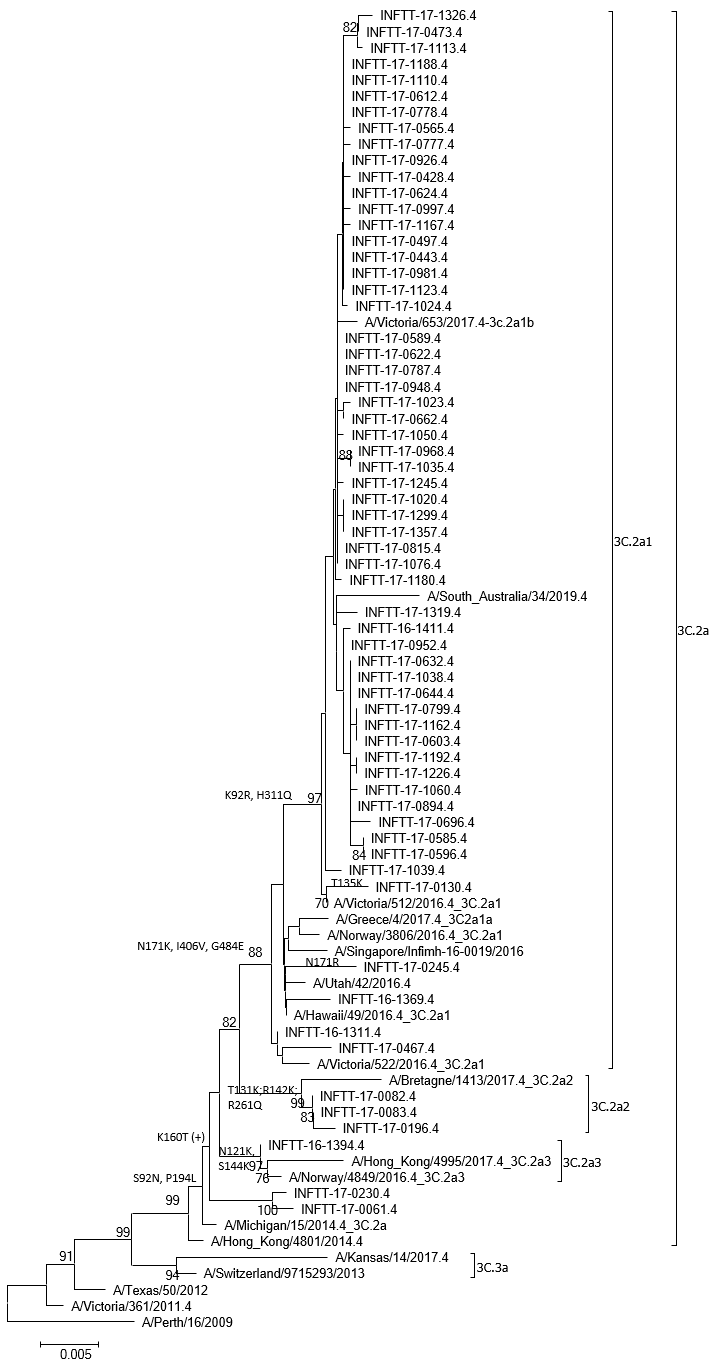
**

**Supplementary Figure 7:** Phylogenetic tree of A/H1 haemagglutinin gene sequenced from a subset of cases.

**
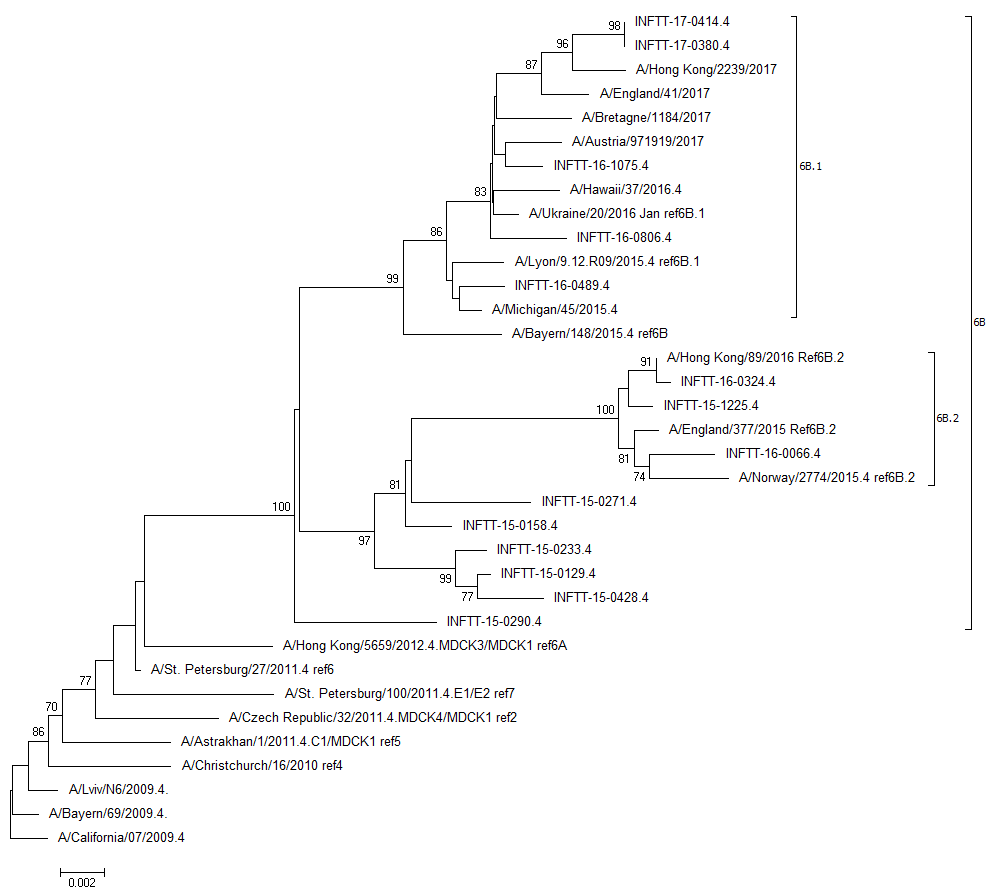
**
